# Evaluation of Anatomic Variations of Fibula Free Flap in Human Fresh Cadavers

**DOI:** 10.29252/wjps.8.2.229

**Published:** 2019-05

**Authors:** Mahdi Gholami, Arya Hedjazi, Amir Kiamarz Milani

**Affiliations:** 1 Department of Oral and Maxillofacial Surgery, Mashhad University of Medical Sciences, Mashhad, Iran;; 2 Iranian Legal Medicine Organization and Research Center for Legal Medicine, Tehran, Iran

**Keywords:** Fibula, Free flap, Fresh cadaver, Skin perforators

## Abstract

**BACKGROUND:**

Reconstruction of the head and neck defects is still one of the most challenging surgeries for the surgeons. This study investigated on anatomic variations of fibula free flap in human fresh cadavers.

**METHODS:**

Twenty fibula free flaps harvested from 10 fresh human corpses were enrolled. The number and type of skin perforators and their origin were recorded during the flap harvesting. After the completion of flap harvesting, the length of vascular pedicle and diameter of the artery and vein at the origin, the fibula length, the distance of the head of fibula to the site of peroneal artery bifurcation and harvesting time were also recorded.

**RESULTS:**

The fibula free flaps were performed on 2 women and 8 men with the mean age of 35.6 years. The average number of perforators per flap was 1.7, most of which were musculocutaneous (35.29%) from soleus muscle. The mean fibula length was 33.1 (range: 31-35) cm. The mean distance of the head of fibula to the site of peroneal artery bifurcation from the tibialis posterior trunk was 5.76 (range: 4.5-6.5) cm. The mean length of the pedicle flap was 11.15 (range: 10-13) cm. The mean diameters of the peroneal artery and vein at the origin were 2.83 and 51.5 mm, respectively.

**CONCLUSION:**

Although the fibula osteocutaneous flap is a reliable choice for maxillofacial reconstruction, flap harvesting is fairly difficult. Accordingly, surgeons must be aware of anatomical variations of the flap and have a suitable case selection to minimize the risk of surgical complications.

## INTRODUCTION

Reconstruction of the head and neck defects is one of the most challenging surgeries for the surgeons working in this field. The goals of the reconstruction of the head and neck defects include the proper restoration of the site, rehabilitation of the sensory and motor activities, and recreation of esthetics.^1^ Since the introduction of the microvascular flap in 1980, these flaps have been identified as the gold standards for the repair of complicated head and neck defects, especially mandibular reconstruction.^2^^,^^3^

The main advantage of micro vascular free flaps is the applicability of these flaps due to their surface texture characteristics, tissue volume, vascular nutrition, and possibility of transmitting the diverse tissues. As a result, defects with different sizes and in different areas can be reconstructed by this flap.^4^ Fibula flap was first used by Taylor *et al.* in 1975; thereafter, in 1979, various techniques were introduced for accessing to the fibula.^5^ This flap is now known as a gold standard for the repair of mandibular defects.^1^

It was shown that septocutaneous arteries represent a small percentage of the perforators that feed the skin paddle, and that most of these perforators are musculocutaneous.^6^ The main advantage of fibula flap is the presence of long bone in the flap that can be used to repair the bone defects. The benefits of this flap include low morbidity, hidden scar, facilitation of access to further tissue, long vascular pedicle, adequate blood supply, ability to simultaneously transfer the skin, bone, and soft tissue along with one-stage vascular anastomosis.^7^^,^^8^

On the other hand, one of the main disadvantages of this flap is its thin tissue that limits its usage in cases with widespread soft tissue loss. Moreover, tissue ischemia and the prohibition of use in people with peripheral vascular disease and venous deficiency and difficult use for less experienced surgeons are other limitations of this flap.^9^^,^^10^ This flap in the head and neck area can be utilized to reconstruct the mandibular region, as well as the scalp, ophthalmic, maxillary and oral soft tissue defects. Considering the aforementioned advantages and limitations, also the extent of anatomical variations in this area, the present study examined the anatomical variation of fibula flap in fresh human cadavers.

## MATERIALS AND METHODS

Our study was performed on 10 fresh human cadavers with no trauma history or congenital or developmental deformities in the lower extremities. A total of 10 free fibula flaps were harvested. The evaluated parameters were (i) Type (namely musculocutaneous, septocutaneous, or septomyocutaneous) and number of the skin perforators in the flap, (ii) Length of the vascular pedicle; measured from the first perforator in the flap (cm), (iii) Artery and vein diameters in the vascular pedicle; measured by a digital caliper (mm), (iv) Distance of each perforators from the fibula head (cm), (v) Distance between the fibula head and the lateral malleolus (cm), and (vi) Flap harvesting time (min).

**Fig. 1:**
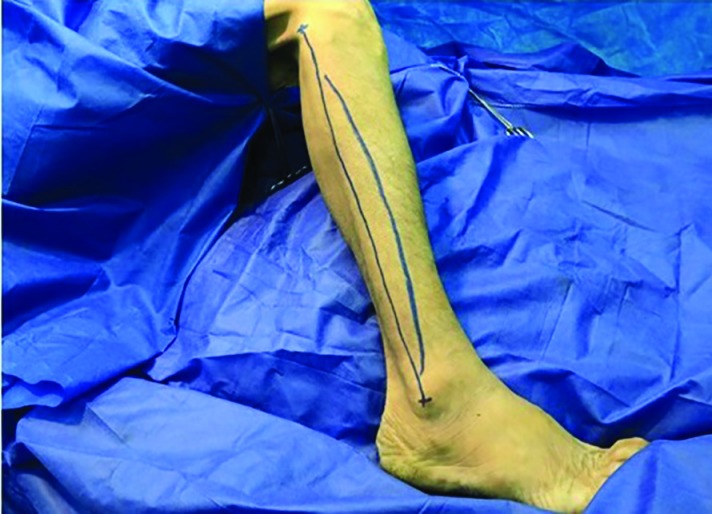
Design of skin island and incision on the right lower lateral extremity

The design of the fibula flap began with drawing a straight line from the lateral fibula head to the lateral epicondyle of the ankle, representing the landmark to the intermuscular septum. Subsequently, anelliptical cutaneous flap was designed from 7 cm below the lateral fibular head to a point 8 cm above the lateral epicondyle of the ankle, centered over the junction of the middle and distal thirds (Figure 1). The skin paddle was incised anteriorly through the skin and subcutaneous tissue overlying the peroneus longus and peroneus brevis muscles. In the next step, an anterior to posterior dissection was preceded in the subfascial plane to reach the skin perforators derived from the muscle or intermuscular septum.

**Fig. 2:**
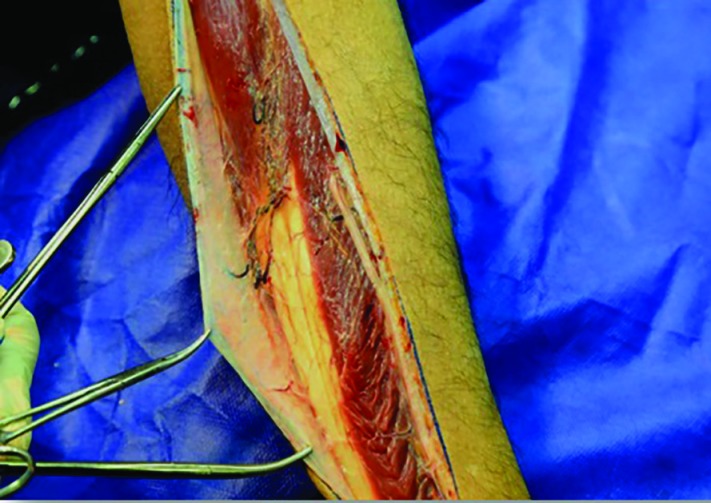
Septocutaneous skin perforators identified coursing out to the skin

**Fig. 3:**
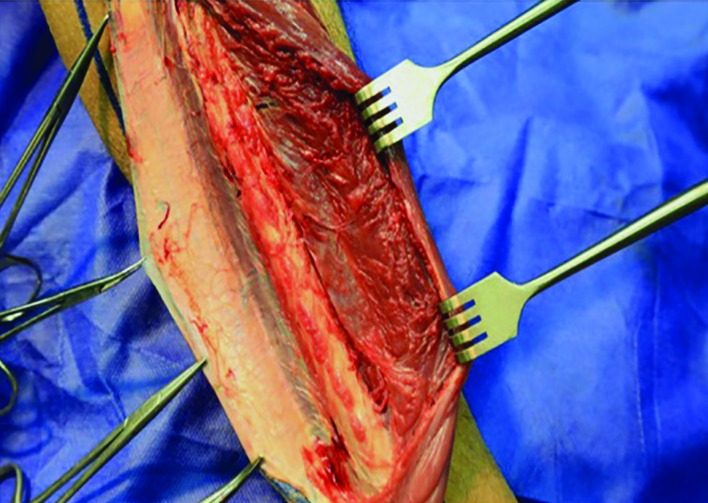
Medial aspect disection of the fibula reveals the inter-osseous membrane

**Fig. 4:**
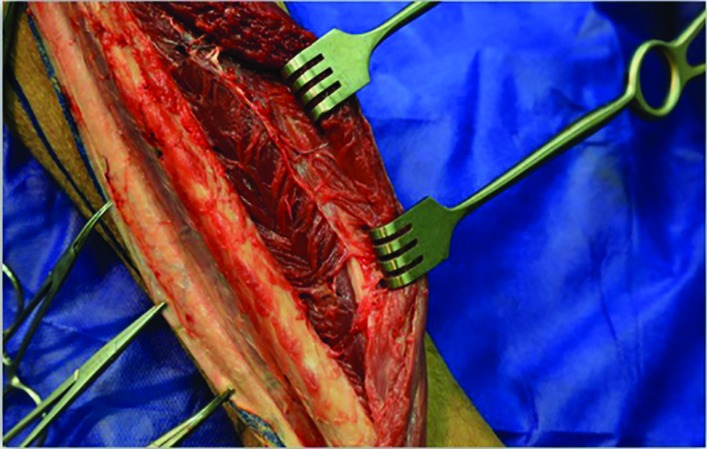
Chevron-oriented muscle fibers of the posterior tibialis muscle (white dashline)

The peroneus longus muscle was retracted medially and the skin paddle was reflected laterally to reveal the posterior crural septum. At this point, the septocutaneous perforators were identified and recorded in the checklist (Figure 2). Further dissection along the anterior aspect of the fibula with elevation of peroneus longus, peroneus brevis, and flexor hallucis longus muscles revealed the inter-osseous membrane (Figure 3). Transection of the inter-osseous membrane revealed the chevron-oriented fibers of the posterior tibialis muscle and facilitated distraction of the fibula (Figure 4). Then the bone cuts were made proximally and distally maintaining a 7 cm bone segment in both sides.

**Fig. 5:**
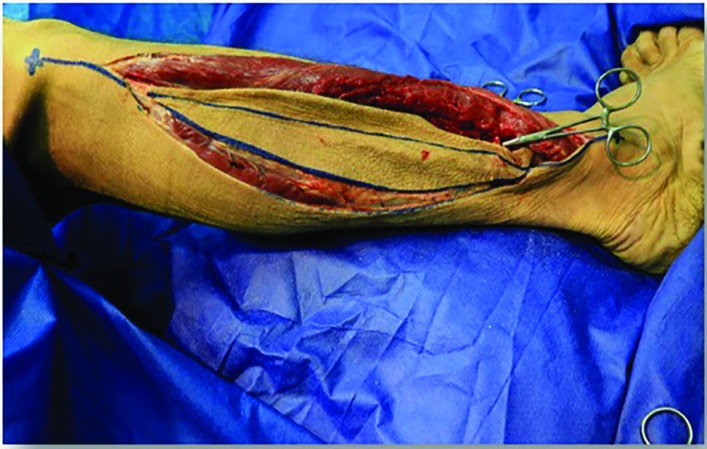
Posterior skin incision of fibula free flap

**Fig. 6:**
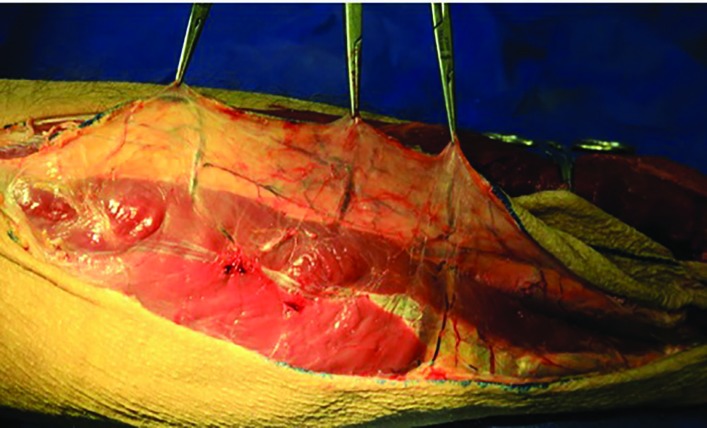
Skin island elevation reveals the musculocutaneous skin perforators

**Fig. 7:**
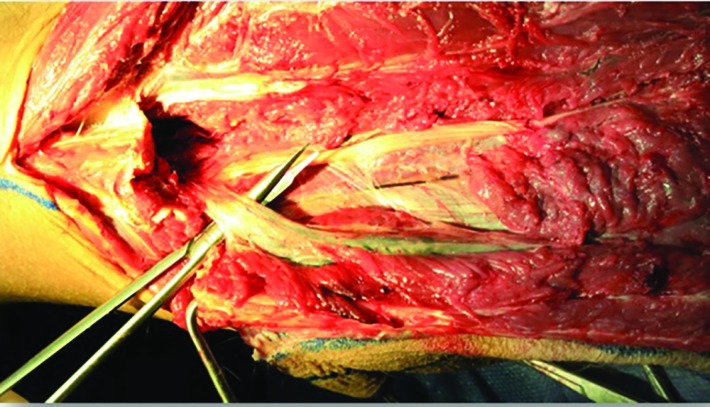
Peroneal vessels disected to the bifurcation of the posterior tibialis vessels

The posterior skin incision was made through the fascia overlying the gastrocnemius and soleus muscles, 7 cm apart from the anterior incision with a fusiform shape (Figure 5). During medial dissection toward the posterior crural septum in the subfascial plane, the musculocutaneous perforators were identified and recorded in the checklist (Figure 6). The gastrocnemius and soleus muscles were transected longitudinally while a 1 cm muscle cuff remained attached to the bone. After distraction of the fibula, the distal end of the proneal artery and vein were identified and transected. Leaving a cuff attached to the flap, the tibialis posterior and flexor hallucis longus muscle fibers were cut and the peroneal vessels were followed proximally to the bifurcation of the posterior tibialis vessels (Figure 7). 

**Fig. 8:**
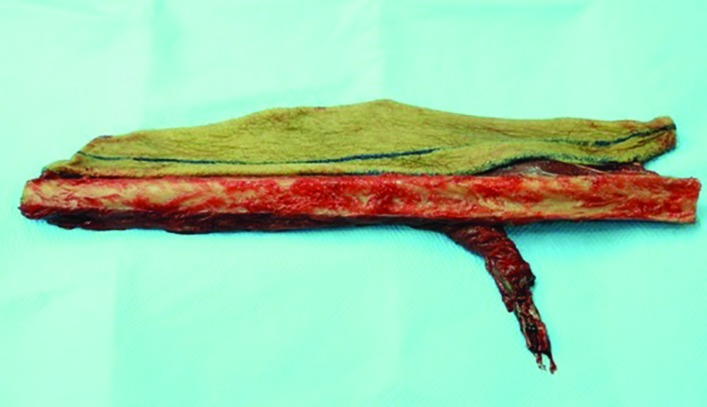
Fibula osteocutaneous flap harvested with peroneal artery and two venae comitantes

At this point the proximal end of the peroneal artery and two venae comitantes were ligated and the fibula osteocutaneous flap was harvested (Figure 8). The time required for the flap harvesting was recorded. The maximum length of the vascular pedicle was measured from the proximal end to the first skin perforator branching site. The diameter of the proximal end of the proneal artery and vein was also measured using a digital caliper. The flap was restored to its place after completion of measurements. Information about the distance of each perforator from the fibula head, the fibula bone length (the distance between the fibula head and the lateral epicondyle of the ankle), age and gender of the subjects were also recorded.

## RESULTS

In this study, 10 cadavers (including 8 male and 2 female cadavers, respectively) were used to assess 20 fibula flaps. The mean age of the bodies was 35.6 years (range from 28 to 45 years). The variables investigated in this study included the number and type of skin perforators, distance of the head of fibula to lateral malleolus (fibula length), distance between the skin perforators and the head of fibula, diameter of the skin perforators, length of the vascular pedicle, the distance of the head of fibula to the site of peroneal artery bifurcation from the tibialis posterior trunk, diameter of the arteries and veins at the origin, and the time of the flap harvesting. 

A total of 20 flaps were made, resulting in the detection of 34 perforators. The mean number of perforators was 1.7 perforators per flap. The maximum number of perforators found was 2 perforators as seen in 14 cases. Out of the total perforators, 12 (35.29%), 6 (17.64%), and 16 (47.05%) cases were musculocutanous, septocutaneous, and septomyocutaneous, respectively (Table 1). Table 2 shows the diameter of skin perforators. The mean diameters of the musculocutaneous and septocutaneous skin perforators were 0.9 and 0.8 mm, respectively. In addition, the mean diameters of the septomyocutaneous skin perforators in the flexor hallucis longus (FHL) and soleus muscles were 1.06 and 1 mm, respectively.

**Table 1:** Number and type of skin perforators in each cadaver

**Cadaver number**	**Number of perforators**	**MC**	**SC**	**SM (FHL)**	**SM (soleus)**
1	4	2	0	2	0
2	2	2	0	0	0
3	4	0	0	2	2
4	2	2	0	0	0
5	4	2	0	2	0
6	4	2	2	0	0
7	4	0	2	0	2
8	4	0	2	0	2
9	2	0	0	2	0
10	4	2	0	2	0
Sum	34	12	6	10	6
Mean	1.7	0.6	0.3	0.5	0.3

MC: musculocutaneous, SC: septocutaneous, SM (FHL): septomyocutaneous in the flexor hallucis longus muscle, SM (soleus): septomyocutaneous in the soleus muscle

**Table 2:** Diameter of skin perforators (mm)

**Cadaver number**	**MC**	**SC**	**SM (FHL)**	**SM (soleus)**
1	0.8	0	1	0
2	1	0	0	0
3	0	0	1	0.9
4	0.7	0	0	0
5	0.9	0	1	0
6	1	0.8	0	0
7	0	0.7	0	1.1
8	0	0.9	0	1
9	0	0	1.2	0
10	1	0	1.1	0
mean	0.9	0.8	1.06	1

MC: musculocutaneous skin perforator, SC: septocutaneous skin perforator, SM (FHL): septomyocutaneous perforatorin the flexor hallucis longus muscle, SM (soleus): septomyocutaneous perforator in the soleus muscle

Table 3 demonstrates the distance between the head of the fibula and the various skin perforators. The mean distance values from the head of the fibula to the first musculocutaneous and septocutaneous perforators were 11.3 (0-13) and 13.3 (0-15) cm, respectively. In addition, the mean distance values from the head of the fibula to the first septomyocutaneous perforator in the FHL and soleus muscles were 18 (0-20) and 22 (0-24) cm, respectively. Table 4 lists the distance from the fibula to the lateral malleolus (fibula length) and the distance between the peroneal artery bifurcation from the tibialis posterior trunk and the fibular head. The mean distance of the head of the fibula to the lateral malleolus was 33.1 (31-35) cm. In addition, the mean distance of the head of fibula to the site of peroneal artery bifurcation from the tibialis posterior trunk was 5.76 (4.5-6.5) cm.

**Table 3:** Distance of fibular head to different skin perforators (cm)

**Cadaver number**	**Fib-MC **	**Fib-SC **	**Fib-SM (FHL) **	**Fib-SM (soleus) **
1	11	0	19	0
2	13	0	0	0
3	0	0	17	24
4	10	0	0	0
5	12	0	16	0
6	10	15	0	0
7	0	14	0	22
8	0	11	0	20
9	0	0	20	0
10	12	0	18	0
mean	11.3	13.3	18	22

Fib-MC: mean fibular head distance to the first musculocutaneous perforator, Fib-SC: mean fibular head distance to the first septocutaneous perforator, Fib-SM (FHL): mean fibular head distance to the first septomyocutaneous perforator in the flexor hallucis longus muscle, Fib-SM (soleus): mean fibular head distance to the first septomyocutaneous perforator in the soleus muscle

**Table 4:** Distance between the fibular head to the lateral malleolus and the site of peroneal artery bifurcation from the tibialis posterior trunk (cm).

**Cadaver number**	**Fib-LM**	**Fib-PTB**
1	32	4.5
2	33	6
3	34	5.3
4	35	5.7
5	31	6
6	31	5.8
7	34	6
8	33	5.3
9	35	6.5
10	33	5.9
mean	33.1	5.76

Fib-LM: mean distance of the fibular head to the lateral malleolus, Fib-PTB: mean distance of the peroneal artery bifurcation from posterior tibialis trunk to the fibula head

Table 5 demonstrates the length of the vascular pedicle, the diameter of the artery and vein in the origin. The mean length of the vascular pedicle was 11.15 (10-13) cm and the mean diameter of the artery at the origin was 2.83 (2.5-3) mm. Furthermore, the mean diameter of the vein at the origin was 3.51 (3-3.8) mm. Table 6 shows the flap harvesting time, according to which the mean harvesting time was 51 (40-65) min.

**Table 5:** Length of the vascular pedicle and diameter of the artery and vein in the origin

**Cadaver number**	**Pedicle length (cm)**	**D-artery (mm)**	**D-vein (mm)**
1	10	2.8	3.5
2	11	2.6	3.3
3	10.5	3	3.8
4	10	3	3.7
5	12	2.5	3.3
6	13	2.9	3.6
7	11	3	3.4
8	12	2.7	3
9	10	3	3.8
10	12	2.8	3.7
Mean	11.15	2.83	3.51

D-artery: mean diameter of the peroneal artery in the origin, D-vein: mean diameter of the peroneal vein in the origin

**Table 6:** Duration of flap harvesting (min).

**Cadaver number**	**1**	**2**	**3**	**4**	**5**	**6**	**7**	**8**	**9**	**10**	**Mean**
Mean time	65	60	55	50	50	50	45	45	40	40	51

## DISCUSSION

Restoration of the head and neck defects has always been a challenging issue for surgeons. In the past, most of these defects were reconstructed by adjacent tissues using pedicle flaps.^11^ Over the past two decades, the use of free microvascular flaps has become widespread. The fibula flap is one of the suitable free flaps to repair the head and neck defects. The use of this flap has been widely accepted in the reconstruction of hard tissue defects, especially the mandibular bone. The implementation of several studies regarding anatomy in this area in recent years has resulted in the promotion of scientists and researchers’ knowledge about the blood flow patterns of this flap.^12^^,^^13^

Accordingly, today, the fibula flap is considered as the gold standard for the treatment of mandibular bone reconstruction. The conventional catheter angiography is a method of choice to identify the main vessels of the leg, anatomical variants of the leg arteries, or other atherosclerotic conditions.^12^^,^^13^ There are also studies suggesting that pre-surgery angiography does not provide enough information.^8^^,^^14^^,^^15^ Other methods, such as CT angiography and magnetic resonance imaging,^16^^-^^19^ have been proposed to characterize the main vascular pedicles to prevent the complications caused by catheter angiography. 

In the past, imaging techniques were not able to recognize the skin perforators; however, these techniques are currently well-improved. Doppler ultrasound is used to detect the location of the skin perforators as a routine process.^20^^,^^21^ Nonetheless, the disadvantage of ultrasound is that it fails to accurately represent the perforators’ location and detect their exact path and origin. In our review regarding the skin perforators of the fibula flap, most of these perforators were musculocutaneous (35.29%), passing through the soleus muscle. 

This was similar to the findings reported before.^3^^,^^22^ In line with other results,^23^ our findings suggested that designing the fibula flap based on the musculocutaneous perforators and maintaining a protective cuff of soleus, FHL and posterior tibialis muscle around the bone, can be an effective method in preserving the flap’s blood flow. However, septocutaneous perforators also account for a small percentage of the fibula skin perforators. Moreover, given their easier dissection, their safety can be more clearly evaluated during and after the flap harvesting in comparison with the musculocutaneous species.^3^^,^^22^ In our study, septocutaneous skin perforators accounted for 17.64% of the perforators.

According to our results, the diameter range of the skin perforators was 0.7-1.2 mm. This is in agreement with the study carried out previously,^24^ which the diameter of the skin perforators in the fibula flap had a mean value of 1.5 mm (range: 0.8-2.3 mm), and another study reported the mean diameter of the musculocutaneous skin perforator at about 1.1 mm.^23^ In our study, the mean distance of the head of the fibula to the first musculocutaneous skin perforators was 11.3 cm. 

In another study,^25^ the mean fibular distance to existing perforators was 10.4 cm. Others,^26^ reported that the mean fibular head distance to the first musculocutaneous and septocutaneous perforator was 12.1 and 18.8 cm, respectively. The difference in the reported distances can be due to differences in the design of the fibula flap, sample size, and race of the subjects studied in the these studies. In our study, the mean length of the vascular pedicle, the diameter of the artery and vein at the origin were 11.15 cm, 2.83, and 3.51 mm, respectively. These diameters facilitated the simple anastomosis of the artery and veins with the vessels of the head and neck during the microvascular surgery. 

There are a few authors examining the diameter of the vessels in the vascular pedicle of the fibula flap as one of the determinants of its suitability for the head and neck anastomosis. In this regard, They had only examined the diameter of skin perforators.^27^ The issue of the vessel diameter may be important as most of researchers addressed microvascular anastomosis to have been carried out on animals vessels at a diameter of 0.8-1.5 mm.^28^ Therefore, the presence of such thick vessels in the fibula flap provides better microvascular surgery given the possibility of convenient anastomosis.

Despite the small number of samples in our research, our results are in line with those of the other studies. However, it is suggested to investigate a greater number of samples and perform new tissue perfusion techniques on human bodies to accustom surgeons with anatomical variations of this flap and acquire more skills in flap harvesting. The results of our study showed that the evaluation of anatomical variation of the fibula flap in the fresh human bodies can be very helpful in better learning the region’s anatomy, improving surgical accuracy, and decreasing harvesting time.

## CONFLICT OF INTEREST

The authors declare no conflict of interest.
